# Staged Management with Transcatheter Aortic Valve Replacement in a Patient with ST-Segment Elevation Myocardial Infarction, Low-Gradient Aortic Stenosis, and Recurrent Cardiogenic Shock: A Case Report

**DOI:** 10.3390/jcm15218218

**Published:** 2026-10-22

**Authors:** Zhongheng Li, Weili Teng, Ruining Li, Liyun He, Dan Li, Lincheng Yang, Mengsha Gao, Ling Xu, Yupeng Wang, Ming Cui, Lijie Sun

**Affiliations:** Department of Cardiology, Peking University Third Hospital, Beijing Key Laboratory of Cardiovascular Receptor Research, Key Laboratory of Molecular Cardiology, Ministry of Education, Key Laboratory of Cardiovascular Molecular Biology and Peptide Modulators, National Health Commission of the People’s Republic of China, Beijing 100191, China; lizhongheng365@163.com (Z.L.); tengweili666@163.com (W.T.);

**Keywords:** aortic stenosis, cardiogenic shock, intra-aortic balloon pump, transcatheter aortic valve replacement, case report

## Abstract

**Background and Clinical Significance:** Cardiogenic shock after successful culprit-vessel revascularization may remain multifactorial when important structural heart disease coexists. **Case Presentation:** We report an 81-year-old man with extensive anterior ST-segment elevation myocardial infarction, low-gradient calcific aortic stenosis (AS) with reduced left ventricular ejection fraction, and cardiogenic shock. Primary percutaneous coronary intervention of a completely occluded proximal left anterior descending artery was performed under intra-aortic balloon pump (IABP) support. Echocardiography showed an aortic valve area of 0.53 cm^2^, a mean transvalvular gradient of 25 mmHg, and a left ventricular ejection fraction of 32%. Although the findings raised concern for a classical low-flow, low-gradient AS phenotype, the low-flow state and true-severe AS could not be formally confirmed because stroke-volume index, dobutamine stress echocardiography, and standardized CT calcium scoring were unavailable. After IABP removal on day 6, profound recurrent shock with lactic acidosis and multiorgan hypoperfusion developed on day 10. IABP reinsertion was followed by improvement in systemic perfusion, whereas brief trial-off periods reproduced hemodynamic and symptomatic deterioration. TAVR was performed on day 24 after recovery of end-organ function, discontinuation of vasoactive drugs, partial platelet recovery, and completion of CT-based procedural planning. IABP was removed the next day, and the patient was discharged on day 30 with an ejection fraction of 42%. **Conclusions:** Recurrent shock after successful culprit-vessel reperfusion should prompt reassessment for persistent structural hemodynamic burdens. Temporary support may serve as a bridge to recovery and individualized procedural planning in selected patients, but the observed improvement after TAVR represents a temporal association in a multifactorial shock state and should not be interpreted as evidence for routine IABP use in infarct-related cardiogenic shock or a predetermined delay before valve intervention.

## 1. Introduction

Cardiogenic shock remains a major cause of early mortality after acute myocardial infarction despite advances in reperfusion therapy and critical care. In patients with ST-segment elevation myocardial infarction (STEMI) complicated by cardiogenic shock, urgent culprit-vessel revascularization is the cornerstone of treatment. However, restoration of epicardial coronary flow does not invariably result in prompt hemodynamic recovery, particularly when severe structural heart disease coexists. Persistent or recurrent shock after successful percutaneous coronary intervention (PCI) should therefore prompt reassessment of additional hemodynamic contributors [1].

Severe aortic stenosis (AS) may substantially aggravate circulatory failure after acute myocardial injury. Fixed left ventricular outflow obstruction limits the ability to augment forward stroke volume, whereas myocardial ischemia, stunning, and infarction reduce contractile reserve. Their coexistence may therefore sustain a low-output state even after successful coronary reperfusion, making the selection of temporary circulatory support and the timing of definitive valve intervention particularly challenging [2].

Contemporary registry data have demonstrated the feasibility of transcatheter aortic valve replacement (TAVR) in selected patients presenting with cardiogenic shock, whereas observational comparisons and a recent meta-analysis suggest that definitive transcatheter valve replacement may be associated with better short-term outcomes than medical therapy or balloon aortic valvuloplasty. Nevertheless, early mortality remains substantial, and available studies do not establish a uniform strategy for temporary mechanical circulatory support or an optimal interval between coronary revascularization and valve intervention [3,4,5].

The present report describes an older patient with extensive anterior STEMI, a low-gradient calcific AS phenotype with reduced LVEF, and recurrent cardiogenic shock. Because confirmatory assessment of low-flow status and true-severe AS was incomplete, the case also illustrates the diagnostic uncertainty encountered in this setting. The clinical focus is the reassessment of persistent structural hemodynamic burden, the selection of temporary circulatory support, and the timing of definitive valve intervention after successful culprit-vessel reperfusion.

## 2. Detailed Case Description

### 2.1. Patient Information and Initial Presentation

An 81-year-old man presented to Peking University Third Hospital (Beijing, China) with persistent chest pain for 4 h accompanied by diaphoresis and fatigue. His medical history included hypertension and type 2 diabetes mellitus. Nine years previously, he had experienced an inferoposterior myocardial infarction attributed to coronary vasospasm and had not continued long-term secondary-prevention therapy.

**Figure 1:**
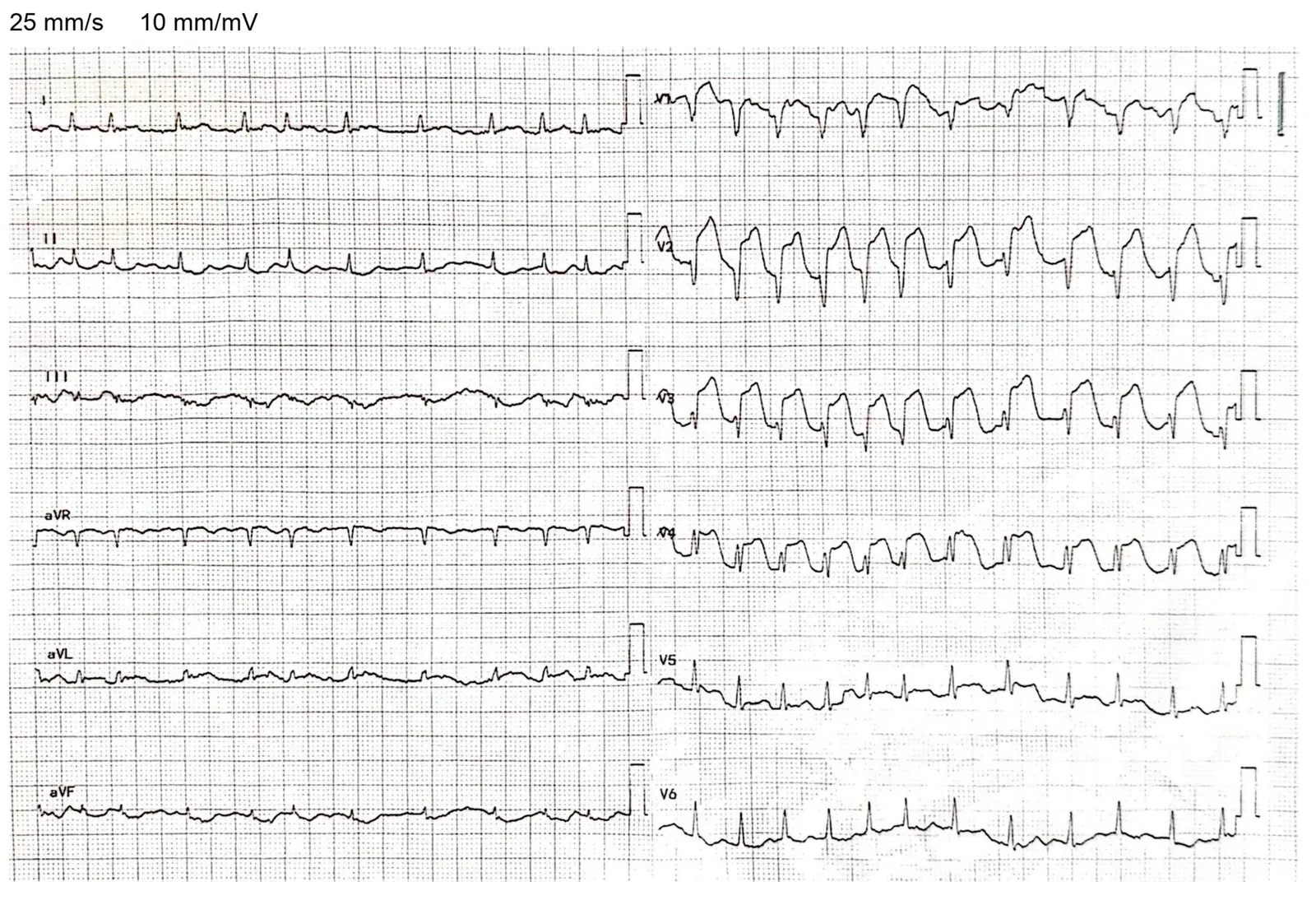
Admission electrocardiogram. A 12-lead electrocardiogram recorded at 25 mm/s and 10 mm/mV demonstrated atrial fibrillation with a ventricular rate of approximately 147 beats/min and marked ST-segment elevation in leads V1–V5, consistent with acute extensive anterior ST-segment elevation myocardial infarction.

On admission, his blood pressure was 85/40 mmHg and heart rate was 147 beats/min. Electrocardiography demonstrated atrial fibrillation with marked ST-segment elevation in leads V1–V5, consistent with extensive anterior STEMI (Figure 1). Initial laboratory testing showed a lactate level of 3.5 mmol/L, troponin I of 0.42 ng/mL, and N-terminal pro-B-type natriuretic peptide of 4840 pg/mL. In view of persistent hypotension and ongoing myocardial ischemia, emergency coronary angiography was performed without delay.

### 2.2. Primary PCI and Evaluation of Aortic Stenosis

**Figure 2:**
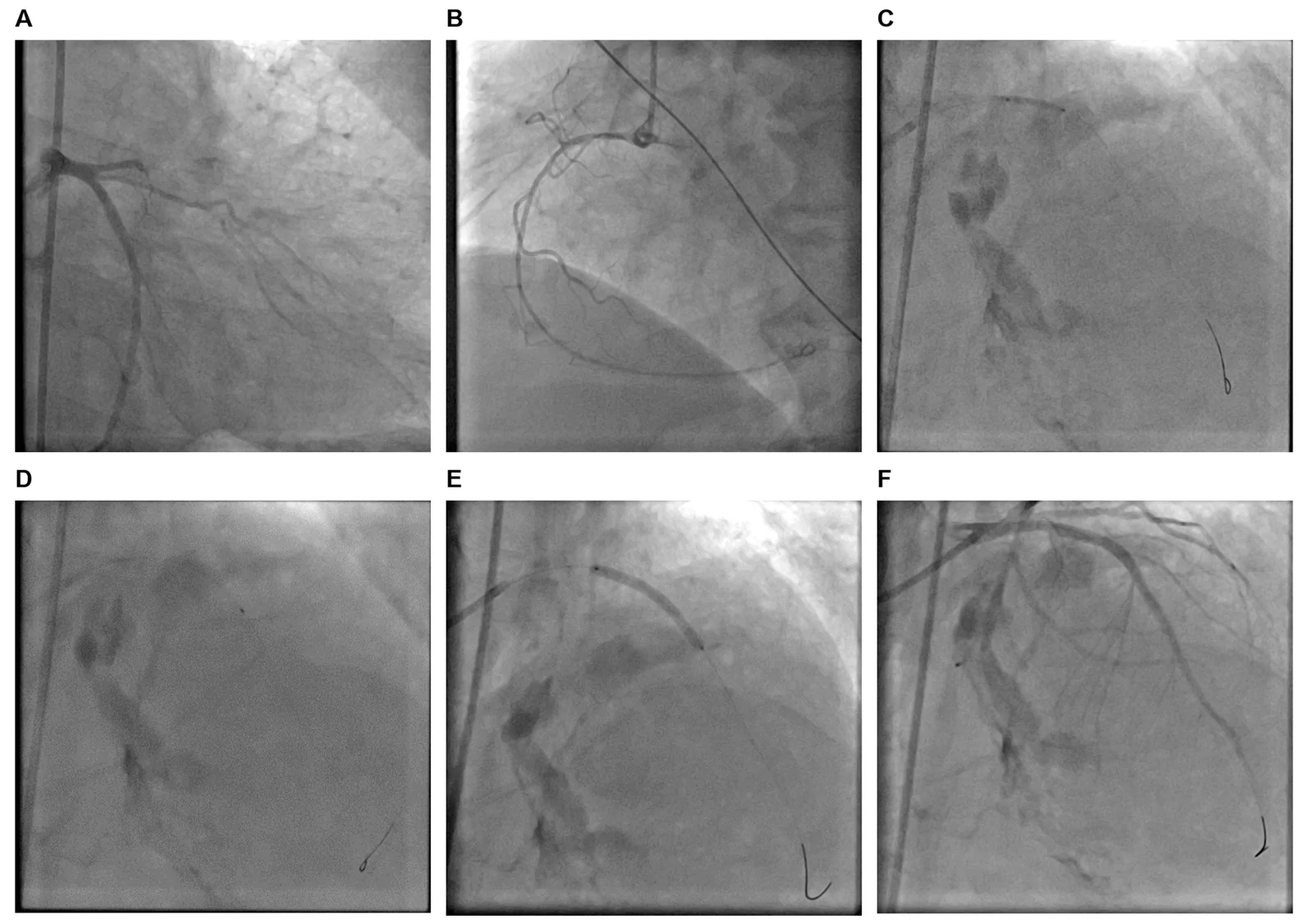
Emergency coronary angiography and primary percutaneous coronary intervention. (**A**) Complete occlusion of the proximal left anterior descending artery. (**B**) Diffuse narrowing of the right coronary artery. (**C**) Balloon dilation of the culprit lesion. (**D**) Thrombus aspiration. (**E**) Drug-eluting stent implantation in the proximal-to-mid left anterior descending artery. (**F**) Final angiographic result after percutaneous coronary intervention.

Coronary angiography demonstrated complete occlusion of the proximal left anterior descending artery (LAD), no significant stenosis in the left circumflex artery, and diffuse narrowing of the right coronary artery (Figure 2A,B). An IABP was inserted through the left femoral artery for hemodynamic support. Initial balloon dilation failed to restore satisfactory antegrade flow; thrombus aspiration was therefore performed, followed by implantation of a 2.75 × 30 mm drug-eluting stent in the proximal-to-mid LAD. Final angiography showed restoration of coronary flow (Figure 2C–F). The patient was subsequently transferred to the intensive care unit with continued IABP and vasoactive support.

Following PCI, the patient’s hemodynamic status gradually improved under continued IABP and vasoactive support. Transthoracic echocardiography on day 1 demonstrated diffuse left ventricular systolic dysfunction with a left ventricular ejection fraction (LVEF) of 32%. The aortic valve was calcified and thickened, with an aortic valve area (AVA) of 0.53 cm^2^, a mean transvalvular gradient of 25 mmHg, and a peak transvalvular velocity of 3.1 m/s. The recorded left ventricular outflow tract (LVOT)/subvalvular peak velocity was 0.69 m/s, yielding a dimensionless peak velocity ratio of approximately 0.22. Serial echocardiography continued to demonstrate a markedly reduced AVA, measuring 0.50, 0.36, and 0.37 cm^2^ on days 6, 12, and 19, respectively, with corresponding mean transvalvular gradients of 31.5, 31, and 39 mmHg. In the setting of AVA ≤ 1.0 cm^2^, mean gradient < 40 mmHg, and LVEF < 50%, these findings represented a low-gradient severe AS phenotype with reduced ejection fraction and raised clinical suspicion for classical low-flow, low-gradient AS. However, stroke-volume index was not available; therefore, the low-flow criterion (stroke-volume index ≤ 35 mL/m^2^) could not be formally confirmed. Dobutamine stress echocardiography was not performed because of the recent extensive STEMI and recurrent hemodynamic instability, and dedicated noncontrast CT calcium scoring in Agatston units was not available. Accordingly, the distinction between true-severe and pseudo-severe AS could not be established by standard multimodality criteria. Nevertheless, the persistently small AVA, low dimensionless velocity ratio, serial gradients reaching 39 mmHg, and markedly calcified valve morphology supported a clinically important fixed valvular obstruction.

### 2.3. Recurrent Cardiogenic Shock and Response-Guided IABP Support

Vasoactive medications were progressively withdrawn and had been discontinued before the initial IABP removal. Counterpulsation remained at a 1:1 ratio, and immediately before removal on day 6, the patient’s blood pressure was 157/75 mmHg with a heart rate of 107 beats/min in atrial fibrillation. Given this apparent hemodynamic stability, IABP was removed. However, progressive dyspnea and worsening heart failure developed over the following several days.

On day 10, the patient developed recurrent cardiogenic shock, with altered mental status, cold extremities, a blood pressure of 80/40 mmHg, and a heart rate of approximately 120 beats/min. Arterial blood-gas analysis showed a pH of 7.267 and a lactate level of 12.52 mmol/L. Serum creatinine increased to 224 μmol/L, accompanied by marked hepatic injury, indicating severe systemic hypoperfusion. Repeat coronary angiography showed no new significant epicardial coronary obstruction. Although this finding did not exclude all possible ischemic mechanisms, it made acute recurrent coronary occlusion less likely as the principal cause of the hemodynamic deterioration.

The differential diagnosis of recurrent shock included persistent post-infarction myocardial dysfunction, recurrent coronary occlusion or other ischemic mechanisms, arrhythmia, infection, and the hemodynamic burden of the coexisting low-gradient AS. Infection was also considered; the white blood cell count was 17.69 × 10^9^/L and procalcitonin was 1.71 ng/mL, and anti-infective therapy with piperacillin sodium/sulbactam sodium was administered. However, the patient remained afebrile, and these inflammatory abnormalities were considered nonspecific in the setting of acute myocardial infarction, profound cardiogenic shock, and systemic hypoperfusion. Serial invasive measurements of cardiac output and filling pressures were not obtained; hemodynamic evolution was therefore assessed using blood pressure, lactate, renal and hepatic function, peripheral perfusion findings, symptoms, and the reproducible response to IABP trial-off.

IABP was therefore reinserted, followed by improvement in blood pressure and end-organ perfusion. After subsequent stabilization, brief trial-off periods of counterpulsation were undertaken to assess whether mechanical support remained necessary. Temporary interruption of IABP support was accompanied by a fall in blood pressure, recurrence of dyspnea, an increase in respiratory rate from approximately 18 to 30 breaths/min, and an increase in heart rate from approximately 100 to 130–140 beats/min. These changes improved after counterpulsation was resumed, a pattern consistent with persistent dependence on temporary mechanical circulatory support.

The recurrent shock was considered multifactorial. Acute myocardial injury, myocardial stunning, severe left ventricular systolic dysfunction, and atrial fibrillation were considered major contributors. However, the persistently small AVA on serial echocardiography, markedly calcified valve morphology, and repeated hemodynamic intolerance during IABP trial-off suggested that the coexisting AS represented an additional fixed hemodynamic burden that could limit forward-flow reserve after coronary revascularization. This interpretation remained probabilistic rather than causal because the low-flow state and true-severe AS were not formally confirmed and myocardial recovery was occurring concurrently.

### 2.4. Recovery, Antithrombotic Management, and TAVR Planning

Given the patient’s age (81 years), recent extensive myocardial infarction, recurrent cardiogenic shock, LVEF of 32%, persistent IABP dependence, and recent renal and hepatic hypoperfusion, the Heart Team considered surgical aortic valve replacement (SAVR) to carry a very high perioperative risk. EuroSCORE II had not been calculated prospectively during the emergency course; using the available preprocedural data and assuming urgent isolated SAVR, it was retrospectively estimated at 29.6%. The Heart Team therefore favored transcatheter aortic valve replacement once a clinically acceptable procedural window could be achieved. Alternative bridge and mechanical-support strategies were considered but were not selected, and VA-ECMO was reserved for periprocedural rescue.

At the time of recurrent shock, proceeding directly to TAVR was considered unfavorable because renal and hepatic dysfunction were still evolving and CT-based annular and access assessment had not yet been completed. During continued IABP support, systemic perfusion and end-organ function progressively recovered: serum creatinine decreased from 224 to 125 μmol/L, alanine aminotransferase from 860 to 74 U/L, and lactate from 12.52 to 0.96 mmol/L; vasoactive medications were no longer required by the day before TAVR. As organ perfusion recovered, CT-based anatomical assessment, valve sizing, access planning, and procedural preparation were completed.

Despite this improvement, the patient remained dependent on IABP, as brief trial-off periods continued to provoke hemodynamic and symptomatic deteriorations. The platelet count decreased from 190 × 10^9^/L to a nadir of 66 × 10^9^/L during critical illness and prolonged temporary support, and subsequently recovered to 116 × 10^9^/L. Testing for heparin-induced thrombocytopenia was negative, and there was no evidence of disseminated intravascular coagulation. By day 24, end-organ function had improved, vasoactive drugs had been discontinued, platelets had partially recovered, and procedural planning had been completed, while IABP trial-off remained poorly tolerated. TAVR was therefore performed at this point rather than after a predetermined waiting interval.

Following PCI, the patient initially received dual antiplatelet therapy with aspirin and clopidogrel. When anticoagulation was added for atrial fibrillation, aspirin was discontinued to avoid prolonged triple antithrombotic therapy, while clopidogrel was retained because of the recent drug-eluting stent. Low-molecular-weight heparin was temporarily withheld at the platelet nadir and resumed after platelet recovery, with clopidogrel maintained through the TAVR period. At discharge, clopidogrel was continued for recent PCI and rivaroxaban was prescribed for long-term stroke prevention in atrial fibrillation.

**Figure 3:**
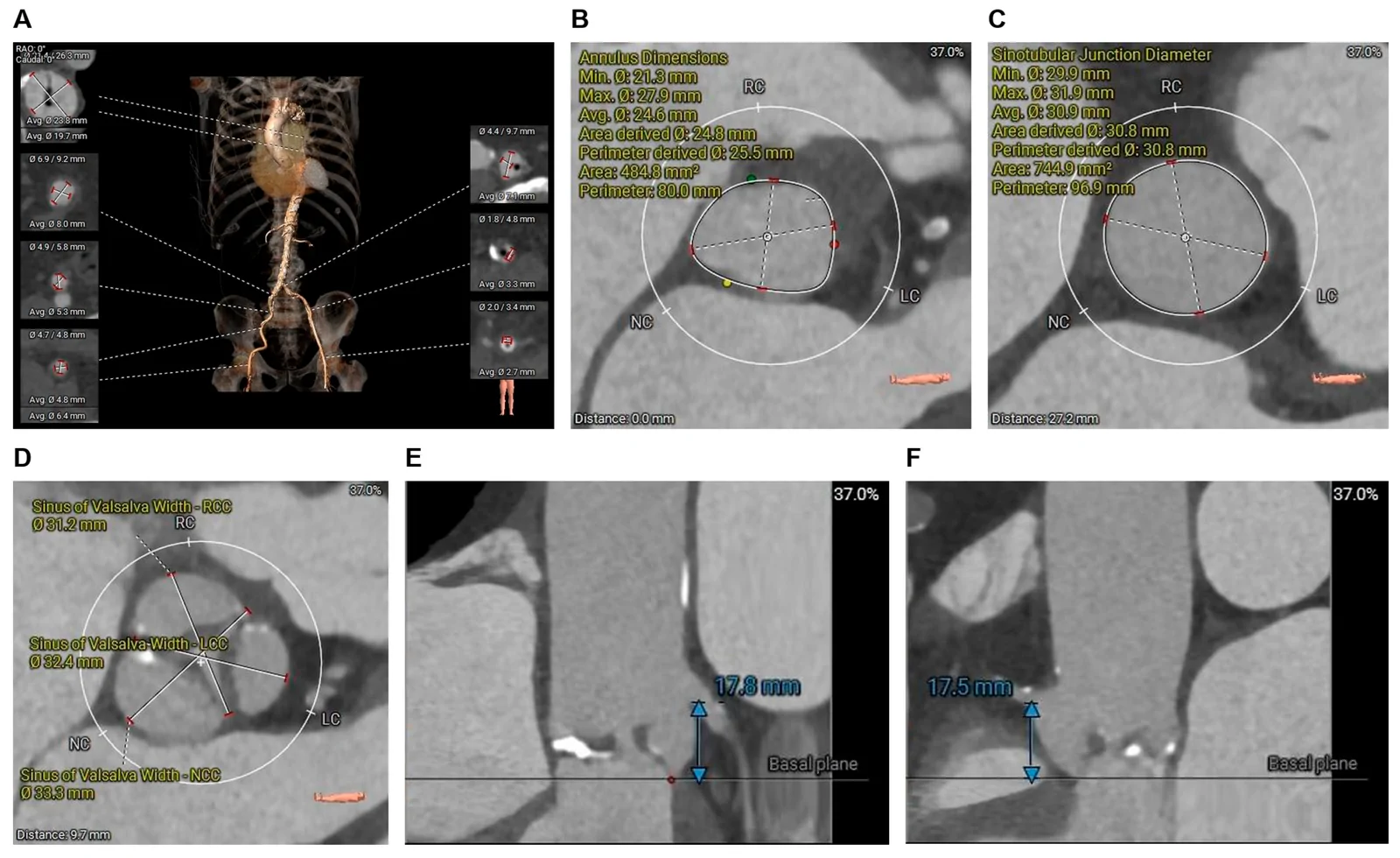
Preprocedural computed tomography angiography assessment before transcatheter aortic valve replacement. (**A**) Transfemoral vascular access. (**B**) Aortic annulus measurement. (**C**) Sinotubular junction measurement. (**D**) Sinus of Valsalva dimensions. (**E**) Left coronary ostial height. (**F**) Right coronary ostial height. LC, left coronary cusp; NC, non-coronary cusp; RC, right coronary cusp. Blue arrows indicate coronary ostial height measurements; other colored markers, lines, and text are software-generated CT workstation annotations used for anatomical measurements.

Preprocedural computed tomography (CT) demonstrated a calcified and thickened tricuspid aortic valve and suitable transfemoral access (Figure 3). The measured total aortic valve calcium volume was 326 mm^3^, predominantly involving the non-coronary cusp; however, dedicated noncontrast calcium scoring in Agatston units was not performed. The aortic annular area was 484.8 mm^2^ and the annular perimeter was 80.0 mm, corresponding to area- and perimeter-derived diameters of 24.8 and 25.5 mm, respectively. The left and right coronary ostial heights were 17.8 and 17.5 mm. The annular plane angle was approximately 60°, consistent with a horizontal aorta and a potential challenge to coaxial delivery. On the basis of the annular dimensions, coronary anatomy, vascular access, and overall clinical condition, transfemoral TAVR with a 26 mm self-expanding transcatheter heart valve was planned.

### 2.5. TAVR Procedure and Follow-Up

TAVR was performed on day 24 under general anesthesia with continued IABP support. Given the patient’s persistent dependence on temporary circulatory support and severely impaired left ventricular function, venoarterial extracorporeal membrane oxygenation (VA-ECMO) was prepared for rapid rescue in the event of periprocedural circulatory collapse. A 26-mm Taurus Max AV self-expanding transcatheter heart valve (Peijia Medical, Suzhou, China) was selected. A corresponding steerable delivery system was used to facilitate coaxial passage across the aortic arch and native valve in the setting of a horizontal aorta.

During advancement of the delivery system, IABP counterpulsation was temporarily interrupted to minimize the risk of interaction between the delivery system and the intra-aortic balloon. Systolic blood pressure decreased transiently to approximately 80 mmHg and recovered after counterpulsation was resumed.

**Figure 4:**
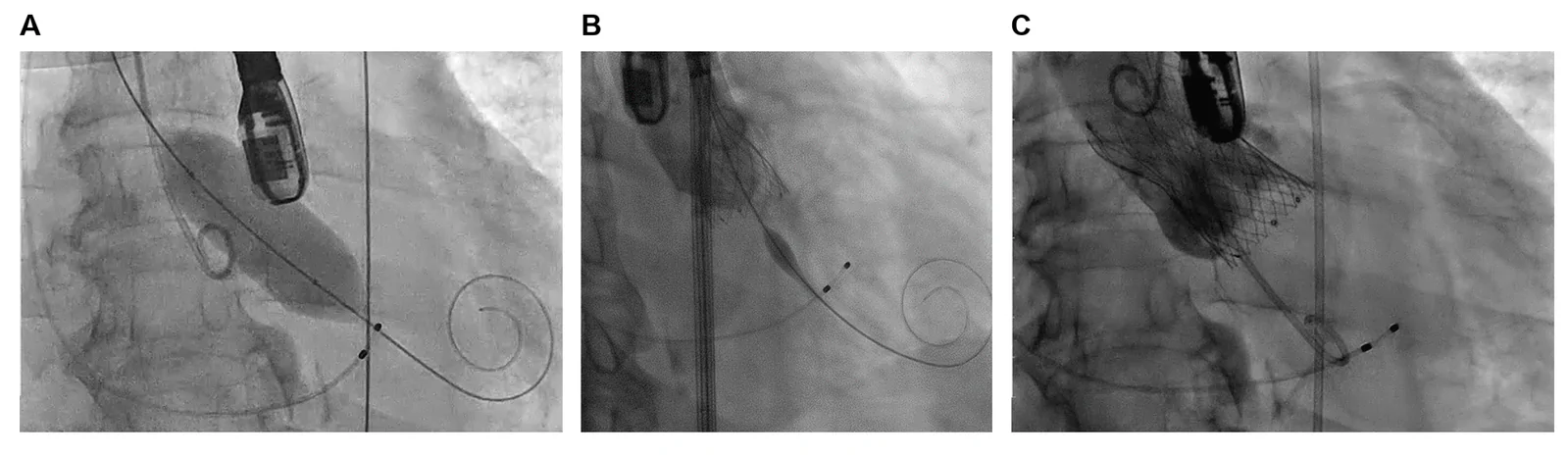
Transcatheter aortic valve replacement procedure. (**A**) Balloon predilation under rapid ventricular pacing. (**B**) Positioning and deployment of the self-expanding transcatheter heart valve. (**C**) Final aortography showing satisfactory valve position and expansion; completion angiographic and echocardiographic assessment showed only trace paravalvular regurgitation.

Following balloon predilation under rapid ventricular pacing, the transcatheter heart valve was advanced and deployed under fluoroscopic and echocardiographic guidance (Figure 4). Multiplanar angiographic assessment confirmed satisfactory valve position and expansion, with preserved coronary flow. Final angiography and echocardiography demonstrated satisfactory prosthetic-valve function with trace paravalvular regurgitation. Postprocedural echocardiography demonstrated satisfactory prosthetic-valve function, with a peak transvalvular gradient of 3.5 mmHg and a mean transvalvular gradient of 1.9 mmHg.

Hemodynamic status remained stable after the procedure, and IABP was removed the following day without recurrent hemodynamic deterioration. This temporal sequence was clinically compatible with improved circulatory reserve after valve intervention, but it cannot separate the contribution of relief of valvular obstruction from ongoing myocardial recovery and other concurrent treatments. The patient continued to recover without recurrent cardiogenic shock or worsening heart failure and was discharged 6 days after TAVR (day 30 after admission). Before discharge, transthoracic echocardiography demonstrated stable prosthetic-valve position, normal leaflet motion, trace paravalvular regurgitation, and improvement in LVEF from 32% to 42%. At discharge, clopidogrel was continued because of the recent coronary stent, while low-molecular-weight heparin was transitioned to oral rivaroxaban for long-term anticoagulation in the setting of atrial fibrillation.

At the 1-month follow-up, the patient remained clinically stable without recurrent chest pain, dyspnea, or heart failure symptoms. Approximately 2 months after discharge, while recuperating at home, he developed pneumonia complicated by respiratory failure and subsequently died.

A concise timeline of the patient’s clinical course and major therapeutic interventions is presented in Table 1.

### 2.6. Patient Consent for Publication

Following his initial discharge, the patient provided written informed consent for publication of his clinical data and accompanying images.

## 3. Discussion

In this patient, assessment of AS severity was complicated by the acute myocardial infarction and markedly reduced LVEF. The initial echocardiogram showed an AVA of 0.53 cm^2^, a mean gradient of 25 mmHg, a peak velocity of 3.1 m/s, an LVEF of 32%, and a dimensionless peak velocity ratio of approximately 0.22; serial studies continued to show a markedly reduced AVA. These findings represented a low-gradient severe AS phenotype with reduced LVEF and raised suspicion for classical low-flow, low-gradient AS. However, stroke-volume index was not available, and neither dobutamine stress echocardiography nor standardized noncontrast CT calcium scoring was performed [2]. The low-flow state and true-severe AS therefore could not be formally confirmed. The persistently small AVA, low dimensionless velocity ratio, and marked valve calcification nevertheless supported the presence of a clinically important fixed valvular obstruction, which was considered together with, rather than separately from, post-infarction myocardial dysfunction. Because transvalvular velocity and gradient are flow dependent, acute myocardial stunning and reduced forward stroke volume may have contributed to the initially low gradients, whereas partial myocardial recovery could have increased measured gradients on serial examinations. No additional quantitative forward-flow measurements were available beyond the reported Doppler parameters. In the emergency setting, these concordant serial findings were therefore used to inform Heart Team judgment despite incomplete formal classification.

IABP was used as temporary, response-guided support rather than as routine therapy for infarct-related cardiogenic shock. Routine IABP use in this setting is not supported by IABP-SHOCK II or current guideline recommendations [1,6]. At recurrent shock, reinsertion of the previously effective IABP was favored because it could be rapidly re-established without crossing the stenotic native valve; transvalvular microaxial support and VA-ECMO had the patient-specific limitations discussed below. In this case, reinsertion was followed by improvement in systemic perfusion, whereas repeated trial-off periods were accompanied by hypotension, dyspnea, tachypnea, and tachycardia. The initial day-6 removal had been based on apparent stability after withdrawal of vasoactive therapy. At that time, the intended strategy was to discontinue temporary support, allow further post-infarction recovery, and reassess the valve lesion for elective intervention once stability was sustained; continued IABP solely to prolong support was not favored because benefit from routine extended use is unproven and device-related risks accumulate with time. In retrospect, the day-10 deterioration showed that apparent stability during full counterpulsation did not necessarily indicate adequate native circulatory reserve. A longer and more closely monitored weaning or trial-off period might have revealed residual support dependence earlier and could have prompted continuation of temporary support or earlier reconsideration of definitive valve intervention. However, the available data do not establish that prolonged IABP support would have prevented the recurrent shock, which was likely multifactorial. After reinsertion, reproducible deterioration during subsequent trial-off attempts provided clearer evidence of support dependence and supported continuation of IABP as a bridge to TAVR. This pattern supported continued temporary circulatory support but did not establish an IABP-specific treatment effect. Prolonged support also required attention to vascular, bleeding, infectious, and hematologic complications. The platelet count fell to 66 × 10^9^/L; heparin-induced thrombocytopenia testing was negative, and there was no evidence of disseminated intravascular coagulation. Antithrombotic therapy was therefore adjusted according to the competing risks of recent drug-eluting stent implantation, atrial fibrillation, thrombocytopenia, and bleeding, with temporary interruption of anticoagulation at the platelet nadir and avoidance of prolonged triple therapy [1].

Other support and bridge strategies were considered but had patient-specific limitations. Balloon aortic valvuloplasty represented a potential temporary bridge, but IABP had already restored sufficient perfusion to allow organ recovery and TAVR planning, so an additional bridging procedure was not pursued [2]. A transvalvular microaxial flow pump would have required crossing the heavily calcified stenotic native valve; a published case using balloon aortic valvuloplasty followed by Impella support as a bridge to TAVR illustrates the anatomy-dependent nature of this approach [7]. VA-ECMO could provide higher-flow rescue but would add bleeding and vascular risk and may increase left ventricular afterload [8,9]. It was therefore reserved for periprocedural rescue rather than instituted pre-emptively.

The timing of definitive valve intervention was determined by the patient’s evolving clinical condition rather than by a planned waiting period. TAVR is feasible in selected patients with cardiogenic shock, but available observational data do not define a uniform interval between coronary revascularization and valve intervention [3,4,5]. In this patient, the Heart Team favored TAVR over SAVR because of advanced age, recent extensive STEMI, recurrent shock, reduced LV function, persistent IABP dependence, and recent multiorgan hypoperfusion. EuroSCORE II had not been calculated prospectively; retrospective reconstruction from the available preprocedural data, assuming urgent isolated SAVR, yielded an estimated operative mortality of 29.6%, consistent with the clinical assessment of high surgical risk.

At the time of recurrent shock on day 10, lactate was 12.52 mmol/L, serum creatinine was 224 μmol/L, and alanine aminotransferase reached 860 U/L, while CT-based annular and access assessment had not yet been completed. With continued support, creatinine decreased to 125 μmol/L, alanine aminotransferase to 74 U/L, lactate to 0.96 mmol/L, vasoactive drugs were discontinued, and the platelet count recovered from 66 to 116 × 10^9^/L. CT-based valve sizing and access planning were then completed. Because IABP trial-off remained poorly tolerated, prolonged temporary support was also becoming less desirable. Day 24 therefore represented the point at which end-organ recovery and procedural readiness were considered sufficient to proceed with TAVR, rather than a predetermined delay.

IABP was removed the day after TAVR without recurrent hemodynamic deterioration, and LVEF had improved from 32% to 42% before discharge. This temporal sequence is clinically relevant but should not be interpreted as proof that relief of AS alone accounted for the improvement; reperfusion-related myocardial recovery, resolution of myocardial stunning, medical optimization, and continued supportive treatment were occurring concurrently. The patient’s subsequent death from pneumonia with respiratory failure approximately 2 months after discharge also limits any inference regarding durable benefit. This case highlights the importance of reassessing persistent structural contributors when cardiogenic shock recurs after apparently successful culprit-vessel PCI. Temporary circulatory support may provide time for stabilization and definitive treatment when immediate intervention is not yet clinically or technically appropriate. The late infectious death also underscores the frailty and persistent non-cardiac vulnerability of elderly survivors of cardiogenic shock, even after successful procedural stabilization.

## 4. Conclusions

Recurrent cardiogenic shock after successful culprit-vessel revascularization should prompt reassessment for persistent structural contributors. In patients with a low-gradient AS phenotype and reduced LVEF, diagnostic uncertainty should be acknowledged when stroke-volume index, dobutamine stress echocardiography, or standardized CT calcium scoring is unavailable. Temporary mechanical support may be used selectively while end-organ function recovers and definitive intervention is planned. In this setting, the timing of TAVR should be individualized according to hemodynamic status, organ recovery, device- and bleeding-related risks, anatomy, and procedural readiness.

## Figures and Tables

**Table 1:** Clinical timeline of major events and therapeutic interventions.

Time	Clinical Events
Day 0	Presentation with extensive anterior STEMI and cardiogenic shock. Emergency coronary angiography demonstrated complete proximal LAD occlusion. Primary PCI with thrombus aspiration and drug-eluting stent implantation was performed under IABP support.
Day 1	Transthoracic echocardiography showed LVEF 32%, AVA 0.53 cm^2^, mean gradient 25 mmHg, peak velocity 3.1 m/s, and a dimensionless peak velocity ratio of approximately 0.22. The pattern was consistent with low-gradient severe AS with reduced ejection fraction and raised suspicion for classical low-flow, low-gradient AS, although stroke-volume index was unavailable and formal low-flow confirmation was not possible.
Day 6	Vasoactive medications had been discontinued. With IABP counterpulsation at 1:1, blood pressure was 157/75 mmHg and heart rate was 107 beats/min in atrial fibrillation. IABP was removed after apparent hemodynamic stabilization.
Days 7–9	Progressive dyspnea and worsening heart failure developed after IABP removal.
Day 10	Recurrent cardiogenic shock occurred with blood pressure 80/40 mmHg, heart rate approximately 120 beats/min, pH 7.267, lactate 12.52 mmol/L, and serum creatinine 224 μmol/L, accompanied by marked hepatic injury. Repeat coronary angiography showed no new significant coronary obstruction, and IABP was reinserted.
Days 10–23	Hemodynamics and end-organ perfusion progressively improved under IABP support. Serum creatinine decreased from 224 to 125 μmol/L and alanine aminotransferase from 860 to 74 U/L. Brief IABP trial-off periods provoked a fall in blood pressure, dyspnea, tachypnea, and marked tachycardia. Platelets fell from 190 to 66 × 10^9^/L and subsequently recovered. CT-based anatomical assessment and TAVR planning were completed.
Day 23	Lactate had decreased to 0.96 mmol/L, vasoactive medications were no longer required, and the platelet count had partially recovered to 116 × 10^9^/L; however, the patient remained dependent on IABP support.
Day 24	Transfemoral TAVR was performed after end-organ recovery, vasoactive-drug independence, partial platelet recovery, and completion of procedural planning, under continued IABP support with VA-ECMO prepared as rescue support.
Day 25	IABP was successfully removed without recurrent hemodynamic deterioration.
Day 30	The patient was discharged with satisfactory prosthetic-valve function, trace paravalvular regurgitation, and improvement in LVEF from 32% to 42%.
1-month follow-up	The patient remained clinically stable without recurrent chest pain, dyspnea, or heart failure symptoms.
~2 months after discharge	While recuperating at home, the patient developed pneumonia complicated by respiratory failure and subsequently died.

## Data Availability

The clinical data presented in this case report are included in the article. Additional de-identified clinical data are not publicly available because of patient privacy but may be available from the corresponding author on reasonable request.
